# Squamous Cell Carcinoma Arising from Inverted Schneiderian Papilloma: A Case Report with Oral Involvement

**DOI:** 10.1155/2014/478092

**Published:** 2014-06-25

**Authors:** Alexandre Simões Garcia, Diego Maurício Bravo-Calderón, Mariana Pisinato Ferreira, Denise Tostes Oliveira

**Affiliations:** Bauru School of Dentistry, Department of Stomatology, Area of Pathology, University of São Paulo, Alameda Octávio Pinheiro Brisolla 9-75, 17012-901 Bauru, SP, Brazil

## Abstract

Inverted Schneiderian papilloma is an uncommon benign tumor that presents tendency to recur and propensity to be associated with malignancy in approximately 10% of the cases. Some of these lesions are isolated in the maxillary sinus, and predominantly affect white males with mean age of 50 years. We report a case of squamous cell carcinoma arising from inverted Schneiderian papilloma in the maxillary sinus extending to the mouth. The patient was submitted to extraction of a maxillary molar tooth four months before the exacerbation of the symptoms of nasal airway obstruction and facial enlargement. Computed tomography scan revealed a sinonasal mass causing opacification of the right maxillary sinus with destruction of the lateral nasal wall and maxillary sinus floor. The patient was referred to an oncology center for treatment and died from tumor progression one year after the cancer was diagnosed. The intention of this report is to alert dentists to include the inverted Schneiderian papilloma, either associated with squamous cell carcinoma, or not, in the differential diagnosis of maxillary sinus tumors with aggressive behavior, which may extend to the oral cavity or involve roots of teeth.

## 1. Introduction

Papillomas are uncommon benign tumors of Schneiderian mucosal origin [1, 2]. Three subtypes of this tumor are recognized: exophytic papilloma, oncocytic papilloma, and inverted papilloma [1, 3–5]. The inverted papilloma representing around 4% of all primary sinonasal tumors is the most common type of Schneiderian papilloma and it occurs predominantly in white males with mean age of 50 years [2, 3, 6].

Despite its benign nature, the inverted Schneiderian papilloma has a high propensity for local recurrence and may undergo malignant transformation into squamous cell carcinomas [2, 3, 7]. Approximately 20% of these tumors may present several degrees of epithelial dysplasia, which makes a malignant potential to the inverted Schneiderian papilloma [8].

The etiology of inverted Schneiderian papillomas remains controversial, but factors such as chronic inflammation, allergy, occupational pollutants, and mainly infection with human papilloma virus have been suggested [2, 4]. Complete surgical resection with a life-long followup for detection of the possible recurrences is the recommended treatment for this tumor [4, 9–12].

We report a case of invasive squamous cell carcinoma that arose from an inverted Schneiderian papilloma, causing maxillary sinus destruction with extension to and involvement of the oral cavity.

## 2. Case Report

**Figure 1:**
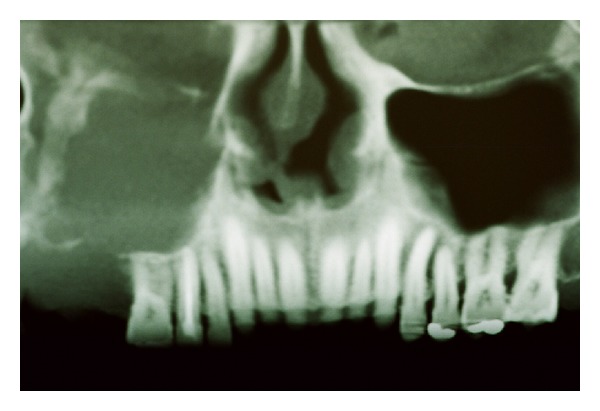
Cone beam computed tomography scan showing a sinonasal mass causing opacification of the right maxillary sinus with erosion of the orbital floor, lateral nasal wall, and maxillary sinus floor.

**Figure 2:**
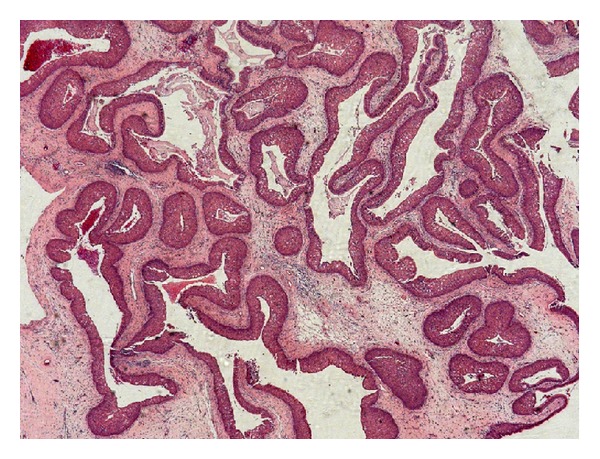
Typical histopathological features of the inverted Schneiderian papilloma showing endophytic growth of pseudostratified ciliated epithelium forming cystic spaces (H.E—25x).

**Figure 3:**
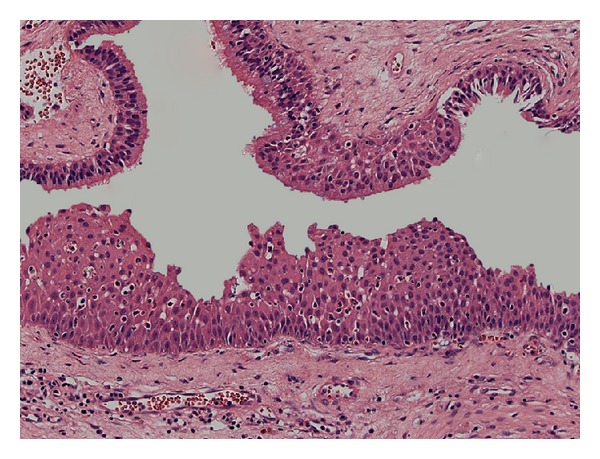
Pseudostratified ciliated epithelium of the Schneiderian mucosa with dysplastic areas (H.E—200x).

**Figure 4:**
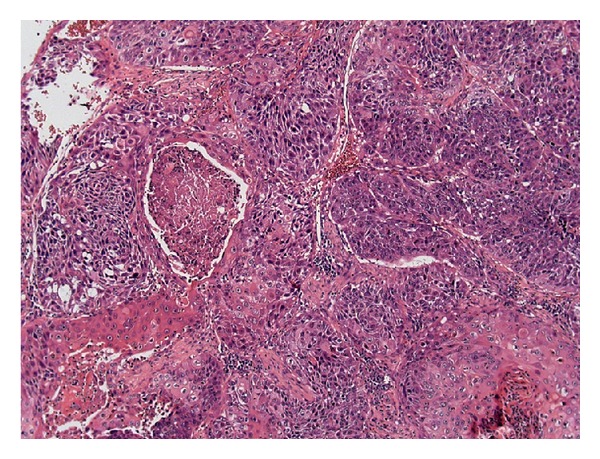
Invasive squamous cell carcinoma showing intense pleomorphism, atypical mitosis, and focal necrosis (H.E—100x).

A 53-year-old man presented with a painful mass in the posterior maxillary alveolar ridge, symptoms of nasal airway obstruction, and facial enlargement. The patient reported a history of tooth extraction (tooth 17) due to a periapical lesion, four months previously. The rest of his medical history was unremarkable. Radiographic evaluation including a computed tomography scan revealed a sinonasal mass causing opacification of the right maxillary sinus with destruction of the lateral nasal wall and maxillary sinus floor (Figure 1). The presumptive diagnosis was aggressive mucocele of the maxillary sinus or malignant tumor. The patient underwent surgical resection under general anesthesia in a hospital, and a friable mass was removed from the maxillary sinus. Gross pathologic examination of the surgical specimen revealed multiple polypoid fragments with the largest diameter being around four centimeters. Histopathological examination revealed endophytic growth of pseudostratified ciliated epithelium admixed with mucous cells forming cystic spaces into an underlying fibromyxoid stroma with an intact epithelium basement membrane, compatible with inverted Schneiderian papilloma (Figure 2). At high-power magnification, squamous metaplasia with variables degrees of dysplasia (Figure 3) and areas of invasive squamous cell carcinoma were also found (Figure 4). It was observed that the pleomorphic squamous epithelial cells had replaced the seromucous glands and ducts in patterns that resembled glandular acini. The final diagnosis established was invasive squamous cell carcinoma arising from inverted Schneiderian papilloma. The patient was referred to cancer reference center for treatment and received combined radiotherapy and chemotherapy. He died one year after cancer was diagnosed.

## 3. Discussion

The inverted Schneiderian papilloma is a benign tumor that presents a tendency to recur and propensity to be associated with malignancy. However, its potential malignant transformation has been described in approximately 10% of the cases [2, 3, 9]. This tumor is most predominantly found in white males with mean age of 50 years [2, 3, 6], epidemiological findings that were confirmed in the present case.

Some symptoms such as nasal obstruction, epistaxis, rhinorrhea, and facial pain or pressure are frequently associated with the occurrence of the inverted Schneiderian papilloma but other inflammatory or neoplastic lesions that affect the maxillary sinus may present similar clinical findings [1, 4, 7]. Our patient showed nasal airway obstruction and facial enlargement and he reported that the lesion was exacerbated after extraction of the second maxillary molar. In addition, the presence of unilateral opacification of the maxillary sinus associated with destruction of the lateral nasal wall and maxillary sinus floor was observed by CT tomography. These radiographic findings are commonly found in inverted Schneiderian papilloma; however, these characteristics alone do not indicate malignant transformation of this tumor [4].

The presumptive clinical diagnosis was aggressive mucocele of maxillary sinus or malignant tumor, based in the imaginologic exams. In fact, the radiographic characteristics including opacification and/or bony destruction of the paranasal sinuses are also found in other benign lesions, such as mucoceles of the maxillary sinuses, nasal polyps, or cholesterol granuloma [4, 13, 14]. For this reason the diagnosis of the aggressive lesion arising in paranasal sinuses should be established based on microscopic features rather than clinical parameters [4, 9].

In the present case the histopathological features were typical of inverted Schneiderian papilloma [2, 9, 11], characterized by inversion of the thickened and multilayered nonkeratinizing squamous epithelium replacing normal respiratory mucosa in the underlying edematous stroma, as illustrated in Figure 2. Mitotic activity, dyskeratosis, and several degrees of epithelial dysplasia were also found in our case, which reinforced the malignant potential of the inverted Schneiderian papilloma. Furthermore, the microscopic analysis of the entire surgical specimen revealed areas of the invasive squamous cell carcinoma (Figures 3 and 4). Thus, the final diagnosis established was squamous cell carcinoma arising from an inverted Schneiderian papilloma. The association between inverted papilloma and malignancy can be metachronous when the malignancy develops at the site of a previously benign inverted papilloma or synchronous when both tumors are diagnosed simultaneously [2, 3, 6], with the latter type being found in the present case.

The most accepted treatment for inverted Schneiderian papilloma is complete surgical resection and due to the aggressiveness, possibility of recurrence, and malignant transformation, long-time followup is recommended [3, 4, 7, 11, 12]. In the cases with malignant transformation a total resection of the lesion and subsequent radiotherapy with or without chemotherapy is the most indicated treatment [2–4]. Our patient was referred to a cancer reference center for treatment and received combined radiotherapy and chemotherapy. He died from tumor progression one year after the cancer was diagnosed. According to the Mendenhall et al. [7], the likelihood of cure, when the inverted Schneiderian papilloma is associated with malignancy, such as the present case, is approximately of 50% and postoperative radiotherapy should be considered for the majority of patients.

Although our patient presented clinical symptoms such as nasal airway obstruction and facial enlargement, he sought a dentist and not a physician. Probably, this occurred because he associated the exacerbation of the symptoms with the extraction of a maxillary molar tooth that had been performed four months earlier. Therefore, in conclusion, the intention of this case reported is to alert dentists to include the inverted Schneiderian papilloma, either associated with squamous cell carcinoma or not, in the differential diagnosis of maxillary sinus tumors with aggressive behavior, which may extend to the oral cavity or involve the roots of teeth.

## References

[1] Cheung FMF, Lau TWS, Cheung LKN, Li ASM, Chow SK, Lo AWI (2010). Schneiderian papillomas and carcinomas: a retrospective study with special reference to p53 and p16 tumor suppressor gene expression and association with HPV. *Ear, Nose and Throat Journal*.

[2] Batsakis JG, Suarez P (2001). Schneiderian papillomas and carcinomas: a review. *Advances in Anatomic Pathology*.

[3] von Buchwald C, Bradley PJ (2007). Risks of malignancy in inverted papilloma of the nose and paranasal sinuses. *Current Opinion in Otolaryngology & Head and Neck Surgery*.

[4] Eggers G, Mühling J, Hassfeld S (2007). Inverted papilloma of paranasal sinuses. *Journal of Cranio-Maxillofacial Surgery*.

[5] Piva MR, Santos Tde S, Martins Filho PR, Kumar PN, Souza LM, Silva LC (2011). Inverted papilloma (Schneiderian papilloma) with involvement of the oral cavity: report of an unusual case. *Anais Brasileiros de Dermatologia*.

[6] Vrabec DP (1994). The inverted Schneiderian papilloma: a 25-year study. *Laryngoscope*.

[7] Mendenhall WM, Hinerman RW, Malyapa RS (2007). Inverted papilloma of the nasal cavity and paranasal sinuses. *The American Journal of Clinical Oncology: Cancer Clinical Trials*.

[8] Suarez PA, Adler-Storthz K, Luna MA, El-Naggar AK, Abdul-Karim FW, Batsakis JG (2000). Papillary squamous cell carcinomas of the upper aerodigestive tract: a clinicopathologic and molecular study. *Head & Neck*.

[9] Perez-Ordoñez B (2009). Hamartomas, papillomas and adenocarcinomas of the sinonasal tract and nasopharynx. *Journal of Clinical Pathology*.

[10] Lee TJ, Huang CC, Chen YW, Chang KP, Fu CH, Chang PH (2009). Medially originated inverted papilloma. *Otolaryngology—Head and Neck Surgery*.

[11] Lawson W, Kaufman MR, Biller HF (2003). Treatment outcomes in the management of inverted papilloma: an analysis of 160 cases. *Laryngoscope*.

[12] Mirza S, Bradley PJ, Acharya A, Stacey M, Jones NS (2007). Sinonasal inverted papillomas: recurrence, and synchronous and metachronous malignancy. *Journal of Laryngology and Otology*.

[13] Marques J, Figueiredo R, Aguirre-Urizar JM, Berini-Ayts L, Gay-Escoda C (2011). Root resorption caused by a maxillary sinus mucocele: a case report. *Oral Surgery, Oral Medicine, Oral Pathology, Oral Radiology and Endodontology*.

[14] Ko M, Hwang C, Kao Y, Lui C, Huang C, Peng J (2006). Cholesterol granuloma of the maxillary sinus presenting as sinonasal polyp. *American Journal of Otolaryngology: Head and Neck Medicine and Surgery*.

